# Structured Reporting in Sleep Medicine

**DOI:** 10.3390/diagnostics15091117

**Published:** 2025-04-28

**Authors:** Katharina Bahr-Hamm, Haralampos Gouveris, Barbara Leggewie, Sven Becker, Friederike Bärhold, Benjamin Philipp Ernst

**Affiliations:** 1 Department of Otorhinolarynoglogy, University Medical Center Mainz, 55131 Mainz, Germany; haralampos.gouveris@unimedizin-mainz.de; 2 Department of Otorhinolaryngology, University Hospital Bonn, 53127 Bonn, Germany; barbara.leggewie@ukbonn.de; 3 Department of Otolaryngology, University Hospital Tübingen, 72076 Tübingen, Germany; sven.becker@med.uni-tuebingen.de (S.B.); friederike.baerhold@med.uni-tuebingen.de (F.B.); 4 Department of Otorhinolaryngology, University Medical Center Frankfurt, 60596 Frankfurt am Main, Germany; b.ernst@med.uni-frankfurt.de

**Keywords:** structured reporting, sleep medicine, obstructive sleep apnea

## Abstract

**Background/Objectives**: Somnological findings are often written as free texts, supported by questionnaires. The quality and structure of free-text reports (FTRs) vary between examiners and specialties, depending on the individual level of expertise and experience in sleep medicine. This study aimed to compare the quality of free-text reports (FTRs) and structured reports (SRs) from somnological consultations in otolaryngology for patients assessed for obstructive sleep apnea (OSA). **Methods**: This study compared free-text reports (FTRs) and structured reports (SRs) from 50 patients with suspected OSA, including medical history, clinical examination findings, and medical letters, all prepared by six examiners with similar experience levels. A web-based approach was used to develop a standardized template for structured somnological reporting. The completeness and time required for both FTRs and SRs were evaluated, and a questionnaire was administered to assess user satisfaction with each reporting method. **Results**: The completeness scores of SRs were significantly higher than those of FTRs (88% vs. 54.2%, *p* < 0.001). The mean time to complete an SR was significantly shorter than that for FTRs (10.2 vs. 16.8 min, *p* < 0.001). SRs had significantly higher user satisfaction compared to FTRs (VAS 8.3 vs. 2.2, *p* < 0.001). **Conclusions**: Compared to FTRs, SRs for OSA patients are more comprehensive and faster. The use of SR is more satisfactory for examiners and supports the learning effect.

## 1. Introduction

Obstructive sleep apnea (OSA) is a common disease. Due to its current estimated prevalence of 23% in women and nearly 50% in men, the public awareness of OSA is steadily increasing [1]. Moreover, OSA, with the nocturnal desaturations and arousals that it causes, is a disease that affects the whole body and multiple organs [2,3]. Patients often report a variety of daytime and nocturnal symptoms, have a long history of sleep-related problems, and seek treatment from a variety of specialists. Sleep-specific medical history and the accurate documentation of upper airway anatomy may be critical to finding the right treatment for an individual patient [4]. The German Sleep Society (DGSM) has developed a checklist to assess the diagnostic findings and the completeness of the treatment recommendations and discharge letter given by society representatives during certification audits of sleep laboratories as part of quality assurance. However, this checklist mainly relates to re-certification audits of already established sleep laboratories and may generally not be readily available to residents in training [5]. Recently, structured reports (SRs) have been adopted by several medical specialties due to emerging evidence that SRs are superior to free-text reports (FTRs) in terms of report quality and inter-rater reliability [6,7,8]. Quite often, examining and referring clinicians prefer SRs because of their accuracy and clarity [9,10,11].

The utilization of SRs could potentially enhance the comprehension of the disease entity and its therapeutic implications [12]. By employing an SR, healthcare professionals can minimize the likelihood of overlooking crucial elements, thereby ensuring a more comprehensive assessment, particularly when conducted by less experienced practitioners [10,13]. Furthermore, due to their standardized format, SRs can be utilized for a meticulous analysis of high-quality scientific data [14].

Despite this, clinicians often express concerns about the rigidity of structured reporting templates and the potential inaccuracy and time-consuming nature of adapting them to specific findings [15]. Implementing a structured reporting approach can greatly enhance clinical examinations that adhere to a well-defined workflow. Consequently, the establishment of a standardized framework for somnological findings through structured reporting holds significant advantages for physicians seeking to enhance their skills in sleep medicine [16].

Furthermore, a prominent feature of sleep medicine is the use of tools to diagnose and report sleep disorders, particularly sleep-related breathing, which have been developed over many years. The use of digital technologies to collect sleep-related data and generate structured reports has become increasingly important in recent years. These technologies include, for example, wearable devices such as actigraphs or smartwatches that can record sleep patterns, movements, and other relevant parameters. These data can then be analyzed in software platforms to generate detailed insights.

This is why it is important to integrate the results of various findings, such as the results of instrumental examinations, sleep-related medical history and established questionnaires, and clinical examination findings. This comprehensive approach ensures a holistic diagnosis and treatment plan that takes all factors into account. Documenting these findings is essential for both the patient and other healthcare providers involved in treatment. The aim of this study was to assess the comprehensiveness, time efficiency, and user satisfaction of template-based structured reports (SRs) compared to free-text reports (FTRs).

## 2. Materials and Methods

### 2.1. Study Design

A comparison was conducted between free-text reports (FTRs) and structured reports (SRs) from sleep medicine consultations. Six physicians specializing in sleep medicine prepared their reports in the format of either an FTR or SR. FTRs were used for 25 patient consultations, while SRs were also applied in 25 cases. The reports included in this study were written by residents without direct supervision from the attending physician. Patients who attend our consultation hours are adults of all ages and genders with suspected or confirmed obstructive sleep apnea of any severity.

### 2.2. Sample Size Calculation

Following previous methodologies, the required sample size was determined considering the expected effect size when comparing the proportion of FTRs achieving at least 80% completeness with that of SRs [6]. Based on prior experience in sleep medicine, it was estimated that 30% of FTRs would meet the 80% completeness threshold, whereas 80% of SRs would reach the same level [6]. Assuming a statistical power of 80% and a significance level of α = 0.05, these parameters resulted in a minimum sample size of 28 patients, with 14 patients in each group.

### 2.3. FTRs and SRs

The department’s standard operating procedure for somnological FTRs, which list key aspects of somnological history, clinical examination, and procedures, was accessible to each resident. For the SRs, a specific template for the structured reporting of somnological findings was developed using internet-based software (Smart Reporting GmbH, Munich, Germany, https://www.smart-reporting.com/de/) by two board-certified somnologists. The template was created using the department’s standard operating procedure for somnological FTRs.

The medical and linguistic content aligns with the latest recommendations of the German guidelines for non-restorative sleep and sleep disorders [17]. The template was designed to cover all common disease entities or conditions in patients with sleep-disordered breathing who regularly present to outpatient sleep clinics.

**Figure 1:**
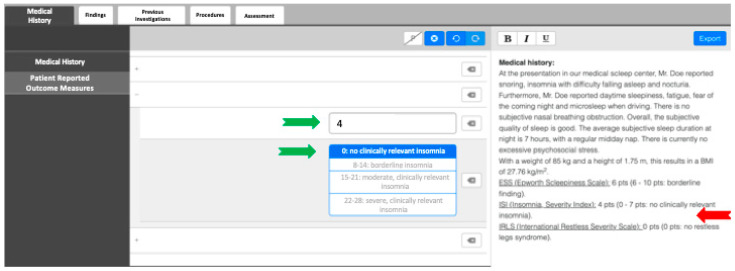
The polysomnographic template with a decision tree. The green arrows show the values entered and clicked in a questionnaire. The text is automatically generated on the right side of the screen. The red arrow shows the section where the questionnaire with the result is mentioned.

In order to create SRs, examiners were provided with clickable decision trees as a guiding tool. The software employed generates fully formed semantic sentences using predetermined text phrases, eliminating the need for further editing (refer to Figure 1). Each report adheres to a consistent structure. For additional flexibility, examiners have the option to include free-text elements as they see fit or to provide additional comments beyond what is required by the template. Additionally, the template includes specific instructions and tutorials, minimizing the need to reference additional medical studies while reporting. All reports were completed promptly by the examining physician immediately following the physical examination and history taking.

### 2.4. Report Evaluation

The time taken to complete each report was recorded during its generation. A total of 50 anonymized reports (25 FTRs and 25 SRs) were independently reviewed for overall completeness (i.e., daytime and nighttime symptoms; sleep questionnaire scores; physical examination of the nasopharynx, oropharynx, and base of tongue; and documentation of external pretest findings such as those from home sleep apnea testing (HSAT) or polysomnography from other sleep laboratories) by a separate physician who was not involved in generating the reports. A specially designed evaluation form was developed by two board-certified somnologists.

### 2.5. User Satisfaction

In addition, we developed a questionnaire for the six examiners. Using a ten-point visual analogue scale (VAS) (10: absolutely agree; 0: strongly disagree), the participating physicians were asked about clarity (question 1); intuitiveness with a short learning curve (question 2); benefits for less experienced physicians being trained in sleep medicine (question 3); efficiency (question 4); whether it was a time saver (question 5); whether any possible additional time required was justified (question 6); improvement in report quality (question 7); usefulness in sleep medicine (question 8); and the clarity of the template layout (question 9), as provided in the Supplementary Materials.

### 2.6. Statistical Analysis

Data are expressed as the mean ± standard deviation, with a *p*-value below 0.05 considered statistically significant. The Wilcoxon signed-rank test was used to assess the significance for completeness and time requirements in paired nominal data. Given the non-parametric nature of the data, the Wilcoxon–Mann–Whitney U test was employed to compare the questionnaire results. All statistical analyses were conducted using Prism 9 (GraphPad Software, Inc., San Diego, CA, USA).

Ethical approval was obtained from the Institutional Review Board (Ethik-Kommission der Landesärztekammer Rheinland-Pfalz. Reference number: 2018-13225; approval date 02/05/2018). All procedures performed in studies involving human participants were in accordance with the ethical standards of the institutional and national research committees and with the Helsinki Declaration of 1964 and its subsequent amendments or comparable ethical standards.

Informed consent for participation is not required as no personally identifiable patient data were included in this study. This study was a retrospective evaluation of a reporting methodology and did not include any personal data. This is in accordance with the Institutional Ethics Committee of the Universities of Mainz and Bonn, Germany (2018-13225 and 374/23-EP).

## 3. Results

### 3.1. Report Analysis

A total of 50 reports (*n* =  25 for FTRs and *n* = 25 for SRs) were eligible for analysis. SRs showed a significantly higher overall completeness (88% vs. 54.2%, *p* < 0.001) and more comprehensive medical history (99.3% vs. 48% *p* < 0.001), findings on clinical examination (86.1% vs. 56% *p* < 0.005), and instrument-based diagnostics (88% vs. 34.2% *p* < 0.001) in the subgroups. The time required for SRs was significantly shorter than that for FTRs (on average 396 s less, 10.2 min vs. 16.8 min, *p* < 0.001); see Table 1.

### 3.2. User Satisfaction

**Figure 2:**
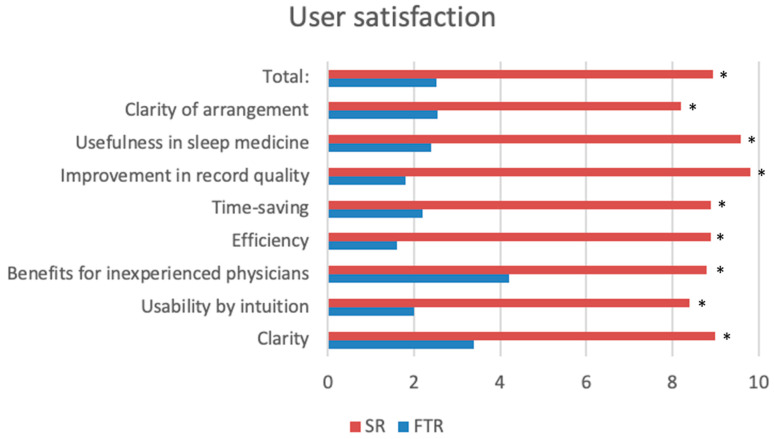
Evaluation of user content with visual analogue scale. * = statistically significant.

The questionnaire results indicated a strong preference for SRs among all surveyed examiners. Overall user satisfaction was significantly higher for SRs compared to FTRs (VAS 8.3 vs. 2.2, *p* < 0.001). Structured reporting was perceived as intuitive and suitable for use in a university medical centre outpatient clinic (8.4 vs. 2.0, *p* < 0.001); it was also considered to be time-efficient (8.9 vs. 2.2, *p* < 0.001) and to have improved documentation quality (9.8 vs. 1.8, *p* < 0.001). In addition, SRs were considered a helpful tool for physicians with less experience in performing somnological examinations (8.8 vs. 4.2, *p* = 0.016). A detailed analysis of the questionnaire results is shown in Figure 2.

## 4. Discussion

In this study, we provided evidence that SRs are beneficial tools in somnological consultations in terms of report completeness, time efficiency, and user satisfaction. Structured reporting can be of great benefit in the context of somnological consultations for OSA by ensuring the standardized and comprehensive documentation of patient information, examination findings, and treatment recommendation. It improves communication between healthcare professionals, facilitates accurate diagnosis, and improves continuity of care. According to the guidelines of the German Sleep Society (DGSM), structured reporting in somnology promotes quality assurance and supports evidence-based practice in the field [18].

Accurate reporting, in addition to comprehensive examination, is critical to maintaining high standards of diagnosis and treatment. Traditional FTRs often lack consistency and reliability in terms of quality, comparability, and the level of detail. However, structured reporting has emerged as a promising alternative, providing a standardized approach that facilitates the generation of comprehensive and reliable reports. This approach aligns with evolving standards in sleep medicine and the goal of improving quality in patient care.

This exploratory, prospective single-centre study aimed to evaluate the feasibility and impact of structured reports (SRs) on somnological recordings for patients suspected of obstructive sleep apnea (OSA), focusing on overall quality, completeness, time efficiency, and user satisfaction. Notably, our study is the first prospective investigation of structured reporting in sleep medicine. Our findings revealed that structured reports (SRs) significantly enhanced report quality and completeness across various subgroups, including medical history, clinical examination, and instrument-based diagnostics. Moreover, disease entities and conditions were described in significantly greater detail, and users reported much higher satisfaction. Importantly, SRs required significantly less time to complete compared to FTRs. These results are consistent with previous studies highlighting the superior report quality achieved with SRs across various diagnostic modalities [10,11,12]. An additional novel finding of this study is that the use of SRs was extremely time-saving (approximately 40%), which directly promotes economic efficiency in sleep medicine, a traditionally personnel-intensive specialty. In addition, both examining and referring physicians expressed a significant preference for SRs due to their standardized approach and alignment with clinical standards and guidelines [14].

As with other specialties, it is likely that SRs in sleep medicine will be useful for educating young residents [19,20]. Patients who seek advice at sleep medical centres, i.e., those with sleep-disordered breathing, present with a variety of rather complex symptoms and often require treatment stemming from various specialties. A complete and comprehensive medical history and an extensive clinical examination are essential for the choice of the right further diagnostic modalities as well as for subsequent treatment decisions. The use of a structured template may provide an added educational value by guiding less experienced residents along the examination path and pinpointing key aspects of the consultation; several publications showing a reduction in missed diagnoses support this argument [19,20,21].

As a monocentric pilot study, this study also has its limitations. On the one hand, although the input mask was designed according to the quality criteria of the DGSM, it was certainly adapted to the individual information requirements of an ENT clinic, so it can be assumed that the input mask in its current form cannot (yet) be transferred to other specialist areas of sleep medicine (e.g., neurology or psychosomatics), since many symptoms to be queried for clinical pictures in these specialties may have been queried only superficially or not at all. It needs to be mentioned that sleep medicine is often considered in different countries as a skill rather than an independent specialty. Thus, it can be practiced by different specialists who have a different focus in sleep medicine, which contributes to the heterogeneity of perceptions. On the other hand, the otorhinolaryngologists who evaluated the questionnaires were all trained at the same sleep medicine centre, so it is not clear whether other otorhinolaryngologists would be able to use the questionnaire to their satisfaction. This could be an impetus for further (multi-centre) studies in the future.

Furthermore, this paper provides a purely descriptive evaluation of the results. FTRs and SRs do not allow any conclusions to be drawn about the quality of treatment or the success of treatment in individual cases. In addition, there was no analysis of the individual condition or mood of the patients in this study.

The debate about medical reporting continues, particularly regarding whether SRs impose overly rigid structures. Some studies suggest that FTRs can produce report quality that is comparable to, or even superior to, that of SRs [15,21]. In addition, SRs have been associated with limitations in linguistic quality, phrasing, and terminology. However, these concerns can be minimized with careful planning. Establishing standardized and recommended language is essential and requires prior discussion between examining and referring physicians to achieve strong consensus and ultimately improve the quality of reporting [22]. Looking to the future, artificial intelligence and advanced technologies offer promising solutions to address these challenges.

In addition, the standardized form of SRs offers an opportunity to collect health data on a large scale for controlling or research purposes (big data). However, it should be kept in mind that sleep medicine is an interdisciplinary field with complex clinical cases and multifactorial influencing factors. In particular, patients with concomitant neurological or psychiatric sleep disorders may have quite individualized histories that are unlikely to be captured by a single template in the future.

## 5. Conclusions

The structured reporting of somnological consultations in ENT departments improves the quality of patient care, time efficiency, and user satisfaction in routine clinical practice. In addition, SRs can contribute to a greater learning effect and can be used for research purposes. Therefore, their implementation in sleep medicine is highly recommended.

## Figures and Tables

**Table 1:** Comparison of free-text reports (FTRs) and structured reports (SRs) in terms of completeness and time required.

	FTR	SR	*p*
Number of reports	25	25	
Overall completeness	54.2%	88%	<0.001
Medical history	48%	99.3%	<0.001
Clinical examination	56%	86.1%	<0.005
Instrument-based diagnostics	34.2%	88%	<0.001
Time required to complete	16.8 min	10.2 min	<0.001

## Data Availability

The data presented in this study are available on request from the corresponding author.

## Supplementary Materials

The following supporting information can be downloaded at https://www.mdpi.com/article/10.3390/diagnostics15091117/s1, Figure S1: Evaluation form of structured recording (SR) of findings in sleep medicine.

## Abbreviations

The following abbreviations are used in this manuscript:

DGSM	German Sleep Society
ENT	Ear–Nose–Throat
FTR	Free-text report
OSA	Obstructive sleep apnea
SR	Structured reporting
VAS	Visual analogue scale
