# Supplementary figures and images for: Structured Reporting in Sleep Medicine
Source: Diagnostics (Basel). 2025 Apr 28;15(9):1117. doi: 10.3390/diagnostics15091117 (PMC12071453; doi:10.3390/diagnostics15091117)

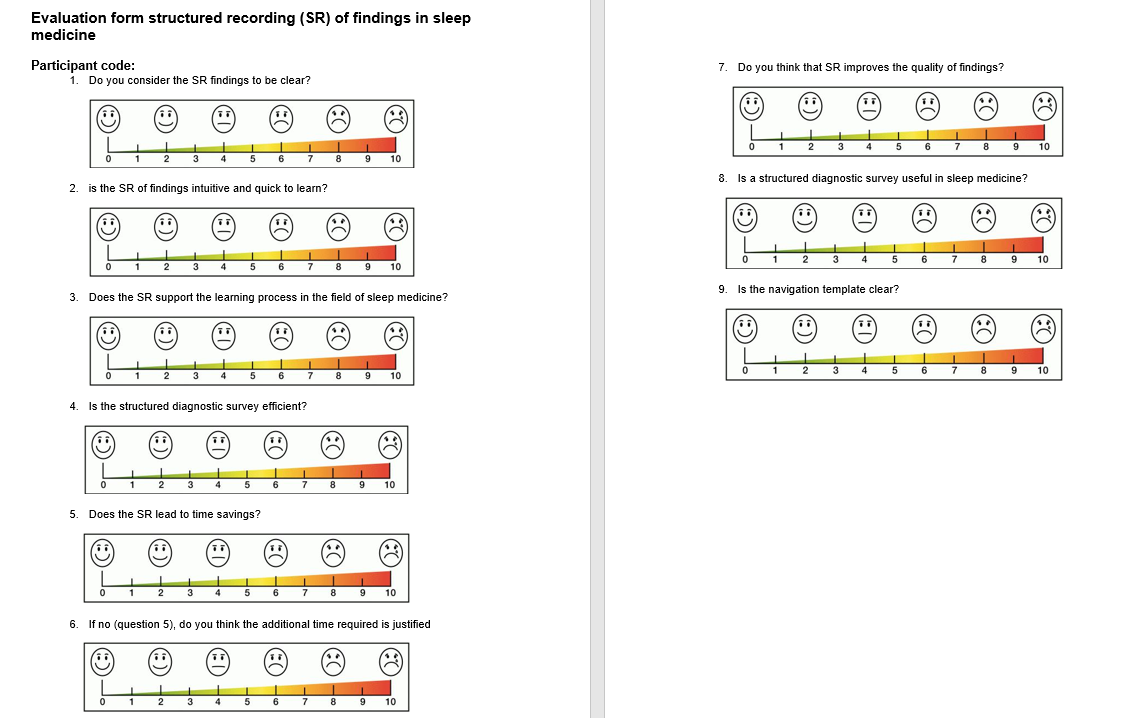

Supplement: Supplementary file 1 [file diagnostics-15-01117-s001.zip › diagnostics-3524175-supplementary.PNG]
